# A Rare Case of Traumatic Coronary Artery Dissection After a Motor Vehicle Collision

**DOI:** 10.7759/cureus.4345

**Published:** 2019-03-29

**Authors:** Mohamed S Elgendy, Toufik Mahfood Haddad, Abhilash Akinapelli, Michael D White

**Affiliations:** 1 Internal Medicine, Creighton University School of Medicine, Omaha, USA; 2 Cardiology, Creighton University School of Medicine, Omaha, USA; 3 Interventional Cardiology, Renown Regional Medical Center, Reno, USA

**Keywords:** coronary artery dissection, blunt chest trauma, cardiac injury, mi, motor vehicle collision

## Abstract

Coronary artery dissection is a rare and life-threatening condition. It can result in thrombus formation and coronary occlusion with subsequent acute coronary syndrome, ventricular arrhythmia, and death. Traumatic coronary artery dissection is an especially rare type of dissection and usually happens in the setting of a high-speed motor vehicle collision. Early recognition and treatment are crucial for survival in patients suffering from this pathology. We present a case of a patient who developed right coronary artery dissection following a motor vehicle collision that was subsequently managed by coronary angiogram and revascularization.

## Introduction

Dissection of a coronary artery is a rare and life-threatening condition. It can result in thrombus formation and coronary occlusion with subsequent acute coronary syndrome, ventricular arrhythmia, and death. Known etiologies include atherosclerosis, trauma, and iatrogenic causes [1]. It may also occur spontaneously with no identifiable cause. Traumatic coronary artery dissection is an especially rare type of dissection and generally happens in the setting of a high-speed motor vehicle collision [2]. Delayed diagnosis of this condition can lead to catastrophic outcomes. Here we discuss a case of traumatic coronary artery dissection.

## Case presentation

A 47-year-old male was brought via ambulance to the emergency department after being struck by another vehicle while driving his motorcycle. His past medical history was significant for non-ischemic cardiomyopathy with ejection fraction of 10%, stage III chronic kidney disease, hypertension, and polysubstance abuse. On presentation, blood pressure was 86/38 mm Hg, pulse 82 beats/min, respiratory rate 22 breaths/min, and oxygen saturation 92% on room air. Primary and secondary trauma surveys revealed Glasgow Coma Scale (GCS) of 7, gross head trauma, and multiple bilateral upper and lower extremity fractures. He was immediately intubated and fluid resuscitation was initiated.

**Figure 1:**
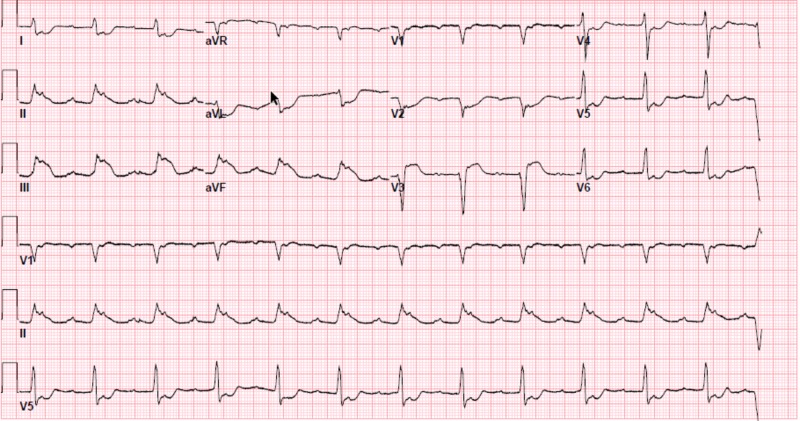
12-lead electrocardiography (ECG) showing sinus rhythm with 2:1 AV block and ST elevation in the inferior leads with reciprocal changes.

Focused Assessment with Sonography for Trauma (FAST) was negative in the right upper quadrant, left upper quadrant, and pelvis. A bedside echocardiogram revealed severe global hypokinesis of both ventricles. While computed tomography (CT) images were being obtained for further evaluation, the patient became hypoxic and bradycardic. Bag mask ventilation was begun, and 0.5 mg atropine was administered. Oxygenation and bradycardia improved, but he then became hypotensive. Subsequently, a right subclavian central venous catheter was inserted, and he was started on inotropes and vasopressors. A 12-lead electrocardiography (ECG) was obtained and showed sinus rhythm with 2:1 AV block and inferior ST elevation myocardial infarction (Figure 1). Serum troponin-I level was found to be elevated at 1.13 ng/mL.

**Figure 2:**
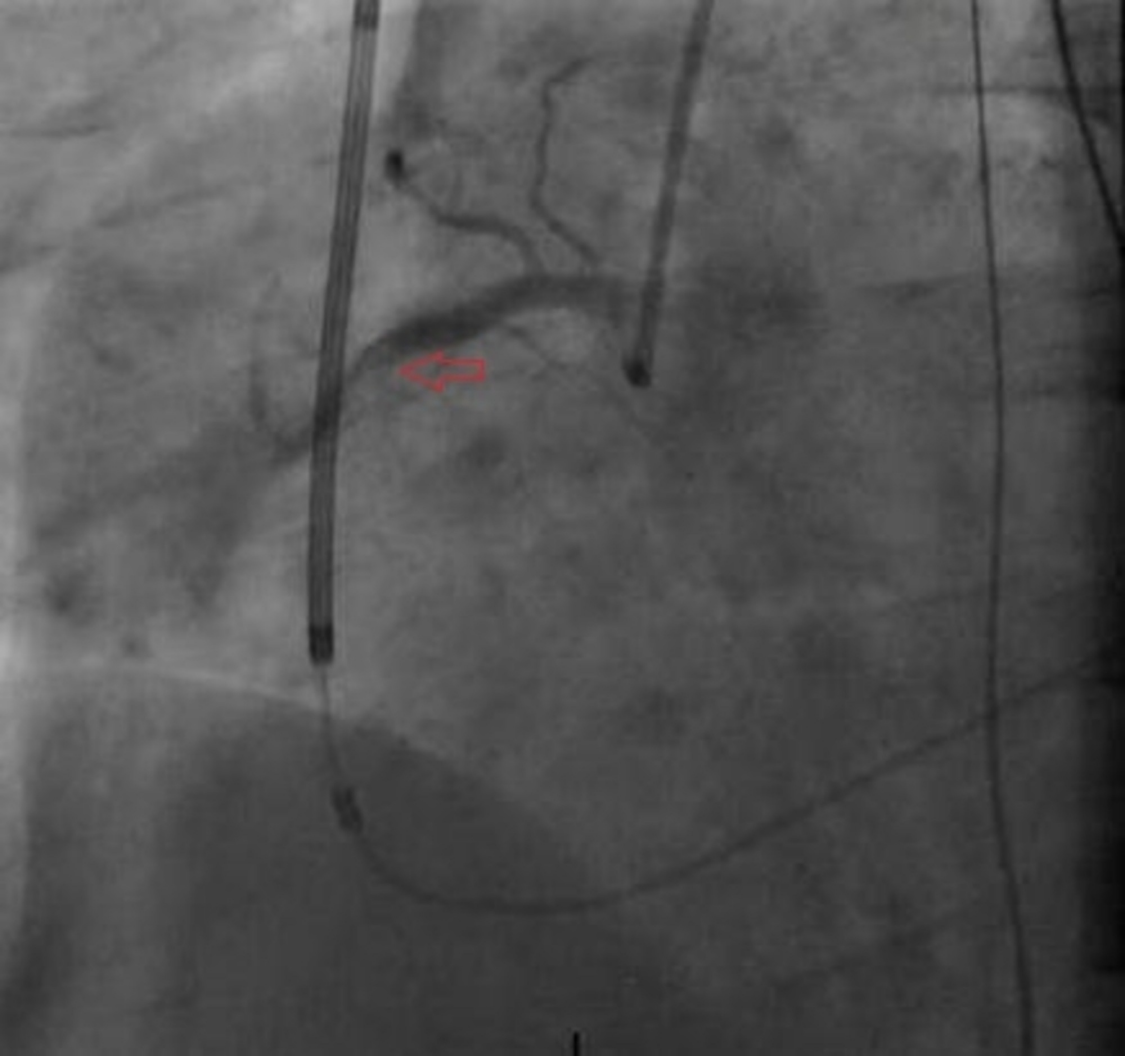
Right coronary artery (RCA) angiography showing total occlusion and suspicious for dissection.

**Figure 3:**
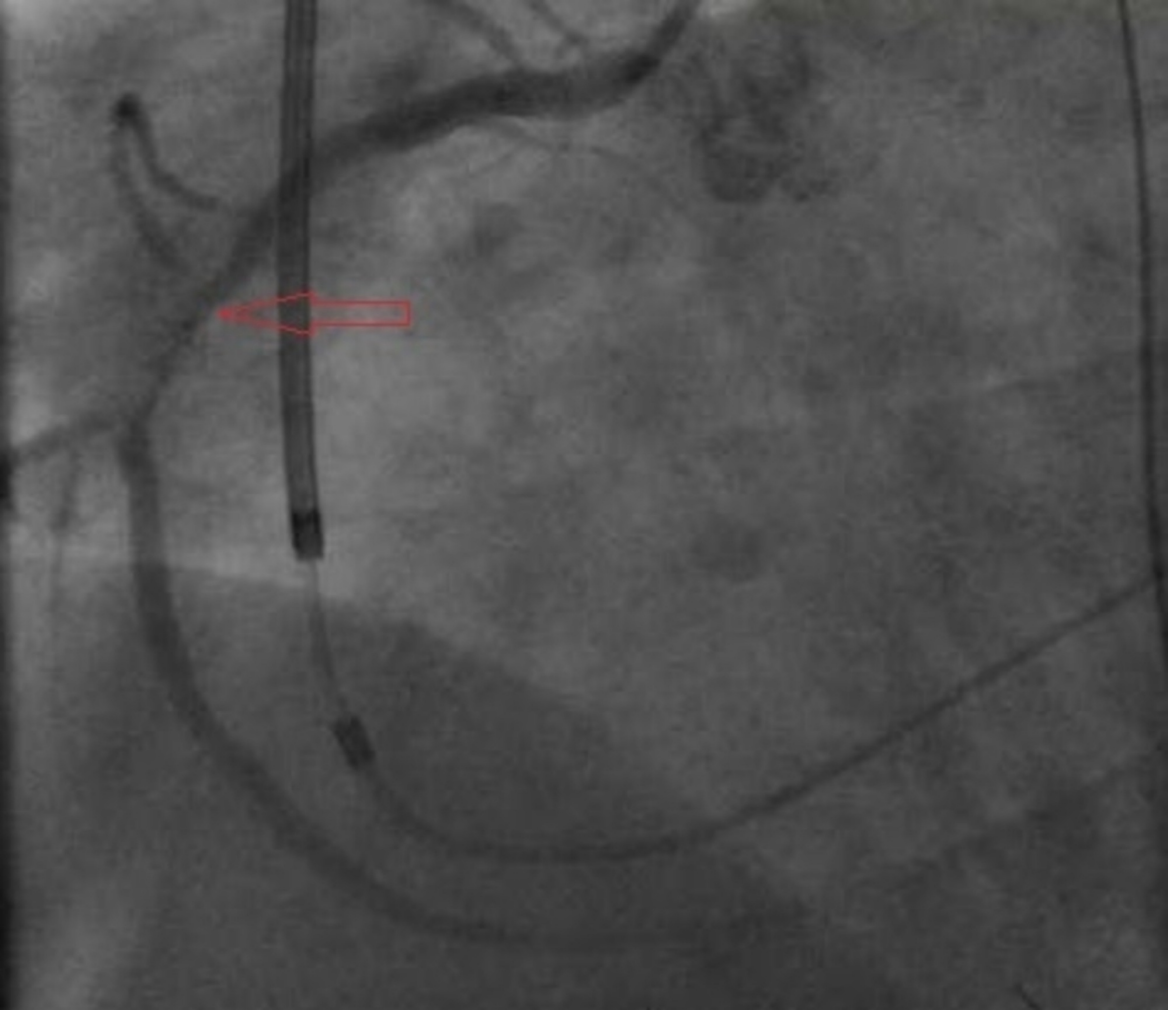
Right coronary artery (RCA) angiography after advancing interventional wire and showing possible dissection flap.

**Figure 4:**
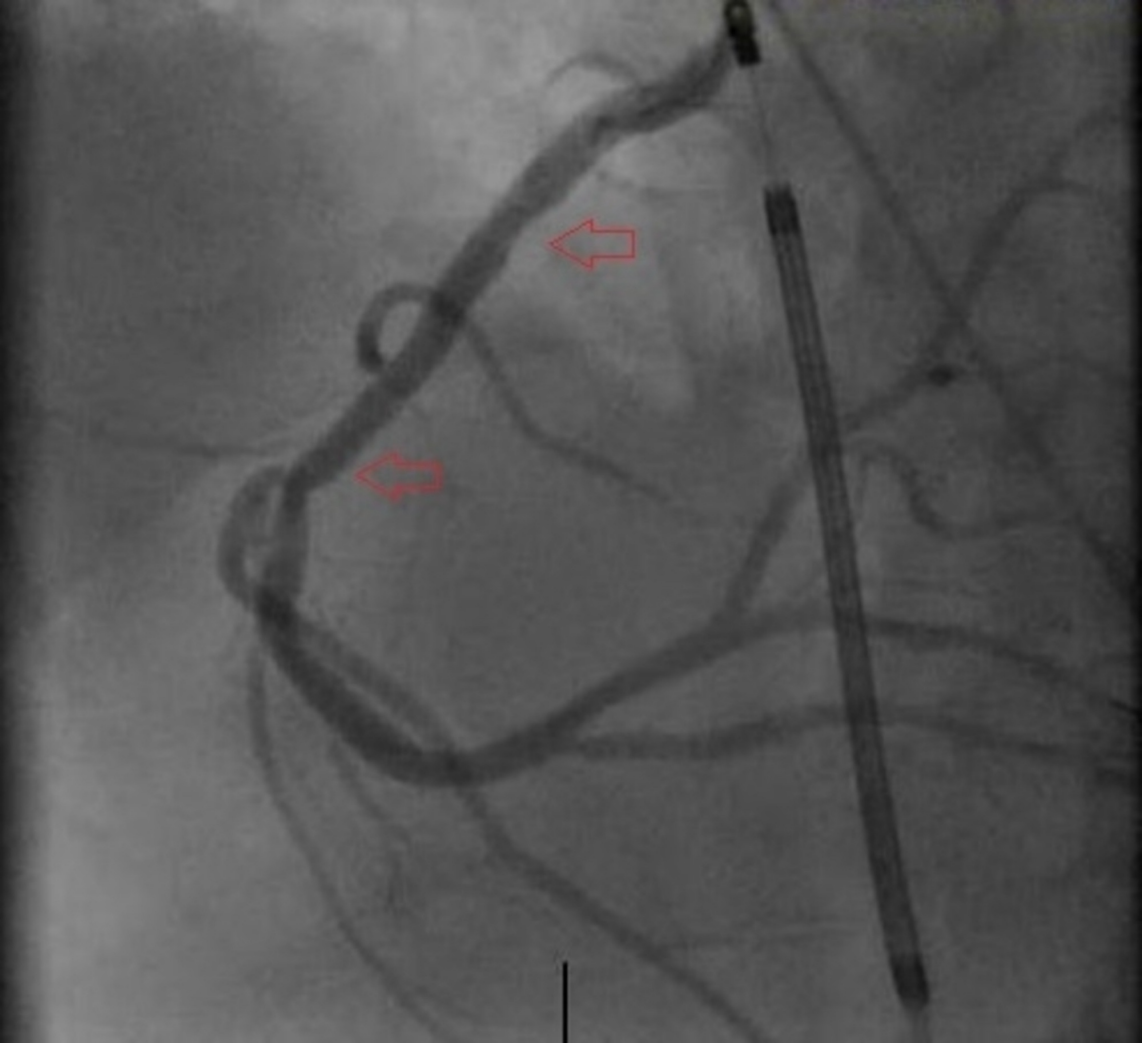
Right coronary artery (RCA) mid segment percutaneous coronary intervention with stent.

The cardiac catheterization lab was activated emergently, and angiography revealed dissection of the mid right coronary artery (RCA) with 100% occlusion (Figures 2-3). Angioplasty was successfully performed with placement of a 4.0 by 28 mm Rebel bare-metal stent in the mid RCA (Figure 4). He was started on aspirin, clopidogrel, and amiodarone and transferred to the intensive care unit for further cares. Unfortunately, the patient continued to suffer from severe decompensated heart failure that progressed to multi-organ failure and ultimately passed away 10 days following admission.

## Discussion

Traumatic coronary artery dissection due to blunt trauma is an extremely rare condition [3]; however, it has been more frequently described in recent literature [4]. This might indicate that this condition has been previously underdiagnosed. It was first described by Kohli et al. in 1988 [5]. High-speed motor vehicle collisions are the number one culprit responsible for blunt cardiac injuries, followed by direct blows to the chest wall, sports-related injuries, and falls [6]. It was even reported that cardiopulmonary resuscitation (CPR) can lead to coronary dissection [7]. Any of the coronary vessels can be affected, however the left anterior descending (LAD) artery is the most commonly involved vessel comprising 76% of cases [8]. The higher incidence of dissection in the LAD artery is thought to be due to its proximity to the chest wall making it more vulnerable to the impact of trauma.

While the definitive diagnosis is most commonly established through cardiac catheterization, it is usually a challenge for physicians to promptly suspect coronary artery dissection because patients may not develop any symptoms until ischemia or infarction develops and also because of its rare occurrence which makes it a distant diagnosis in any clinicians’ mind. A combination of normal ECG and troponin-I at admission and at eight hours has a 100% negative predictive value for significant cardiac injury [9]. Typically, coronary artery dissection would manifest on ECG as hyperacute T waves, which subsequently evolves into ST segment elevations, and eventually formation of Q waves.

Various management strategies of coronary artery dissection have been reported, including percutaneous coronary intervention (PCI), coronary artery bypass grafting, or conservative approach [2].

In our case, the patient had advanced second-degree AV block and ST elevation myocardial infarction. Emergent coronary angiogram was indicated and performed in a timely manner. PCI with stenting of the RCA was a successful approach and indicated with 100% total occlusion of the mid RCA.

## Conclusions

Coronary artery dissection is one of the potentially fatal sequelae of motor vehicle collisions or any kind of blunt chest trauma. Prompt diagnosis and intervention of this condition is critical for a favorable outcome. Because of its rarity, clinical suspicion remains a challenge. One strategy to avoid delay in diagnosis is to routinely obtain ECG and cardiac enzyme levels in patients with blunt chest trauma, however, this strategy requires more research to evaluate its efficacy.
